# Diisopropyl­ammonium thio­cyanate

**DOI:** 10.1107/S1600536812007349

**Published:** 2012-02-29

**Authors:** Jie Xu

**Affiliations:** a College of Chemistry and Chemical Engineering, Southeast University, Nanjing 210096, People’s Republic of China

## Abstract

In the title mol­ecular salt, C_6_H_16_N^+^·NCS^−^, the cation possesses approximate local twofold rotation symmetry. One of its NH atoms forms a hydrogen bond to a thio­cyanate N atom and the other to a thio­cyanate S atom. This results in [001] chains of alternating cations and anions.

## Related literature

For background to mol­ecular ferroelectrics, see: Fu *et al.* (2011 ▶).
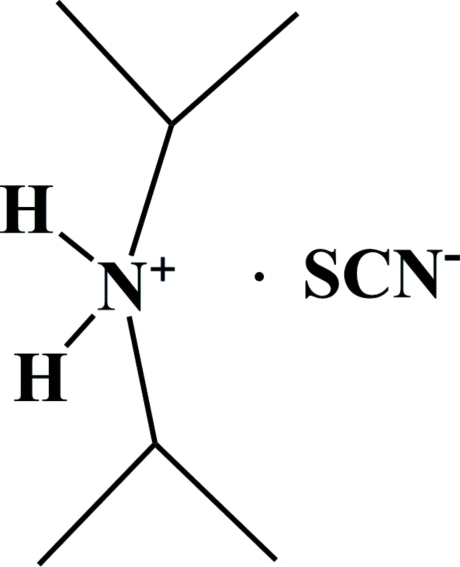


## Experimental

### 

#### Crystal data

C_6_H_16_N^+^·NCS^−^
*M* *_r_* = 160.28
Orthorhombic, 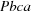

*a* = 11.785 (2) Å
*b* = 12.716 (3) Å
*c* = 13.288 (3) Å
*V* = 1991.3 (7) Å^3^
*Z* = 8
Mo *K*α radiation
μ = 0.27 mm^−1^
*T* = 298 K
0.20 × 0.05 × 0.05 mm

#### Data collection

Rigaku Mercury2 CCD diffractometer
Absorption correction: multi-scan (*CrystalClear*; Rigaku, 2005 ▶) *T*_min_ = 0.90, *T*_max_ = 1.00
19260 measured reflections
2286 independent reflections
1864 reflections with *I* > 2σ(*I*)
*R*_int_ = 0.051

#### Refinement

*R*[*F* ^2^ > 2σ(*F* ^2^)] = 0.045
*wR*(*F* ^2^) = 0.107
*S* = 1.12
2286 reflections
96 parameters
H-atom parameters constrained
Δρ_max_ = 0.18 e Å^−3^
Δρ_min_ = −0.15 e Å^−3^

### 

Data collection: *CrystalClear* (Rigaku, 2005 ▶); cell refinement: *CrystalClear*; data reduction: *CrystalClear*; program(s) used to solve structure: *SHELXS97* (Sheldrick, 2008 ▶); program(s) used to refine structure: *SHELXL97* (Sheldrick, 2008 ▶); molecular graphics: *SHELXTL* (Sheldrick, 2008 ▶); software used to prepare material for publication: *SHELXTL*.

## Supplementary Material

Crystal structure: contains datablock(s) I, global. DOI: 10.1107/S1600536812007349/hb6639sup1.cif

Structure factors: contains datablock(s) I. DOI: 10.1107/S1600536812007349/hb6639Isup2.hkl

Supplementary material file. DOI: 10.1107/S1600536812007349/hb6639Isup3.cml

Additional supplementary materials:  crystallographic information; 3D view; checkCIF report

## Figures and Tables

**Table 1:** Hydrogen-bond geometry (Å, °)

*D*—H⋯*A*	*D*—H	H⋯*A*	*D*⋯*A*	*D*—H⋯*A*
N2—H2*D*⋯N1^i^	0.90	1.99	2.888 (2)	172
N2—H2*E*⋯S1	0.90	2.47	3.3490 (14)	166

Symmetry code: (i) 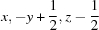.

**Fig. 1.**
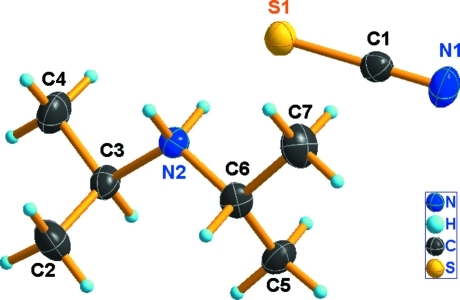
A view of the title compound with displacement ellipsoids drawn at the 30% probability level.

**Fig. 2.**
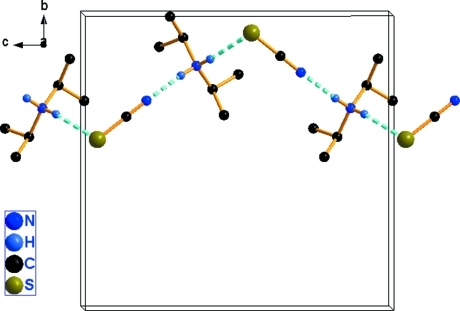
The crystal packing of the title compound showing the one-dimensionnal hydrogen bondings chain along the c axis (dashed line). H atoms not involved in hydrogen bonding (dashed line) have been omitted for clarity.

## supplementary crystallographic information

### Comment

Simple organic salts containig amino cations have attracted attention as materials that display ferroelectric-paraelectric phase transitions (Fu *et al.*, 2011). As part of our ongonig studies in this area, we now present the crystal structure of the title compound, di-isopropylammonium thiocyanate.

The asymmetric unit of the title compound contains one di-isopropylammonium cation and one SCN^-^ anion (Fig. 1). The amine N2 atom was protonated. The SCN^-^ anion is almost linear with the bond angle of 179.80 (2)°. The geometric parameters of the title compound are in the normal range.

In the crystal structure, all the ammonium H atoms are involved in intermolecular N—H···N and N—H···S H-bonding interactions with the N and S atoms of the SCN^-^ anion (with N···N and N···S distances of 2.888 (2)Å to 3.349 (2)Å, respectively). These hydrogen bonds link the ionic units into a one-dimentional chain along the *c*-axis (Table 1 and Fig.2).

### Experimental

A mixture of di-isopropylamine (0.8 mmol), HCl (0.8 mmol) and KSCN (0.8 mmol) were dissolved in EtOH/distilled water (1:1 *v*/*v*) solvent. The solution was slowly evaporated in air affording colourless blocks.

### Refinement

All H atoms attached to C atoms were fixed geometrically and treated as riding with C-H = 0.98 Å(C methine) and C-H = 0.96 Å(C methyl) with *U*_iso_(H) = 1.2*U*_eq_(C methine) and *U*_iso_(H) = 1.5*U*_eq_(C methyl). The positional parameters of the H atoms (N) were refined freely. And in the last stage of the refinement, they were restrained with the H—N = 0.90 (2)Å, and *U_iso_*(H)=1.2*U_eq_*(N).

### Crystal data

**Table:**

C_6_H_16_N^+^·NCS^−^	*F*(000) = 704
*M**_r_* = 160.28	*D*_x_ = 1.069 Mg m^−^^3^
Orthorhombic, *P**b**c**a*	Mo *K*α radiation, λ = 0.71073 Å
Hall symbol: -P 2ac 2ab	Cell parameters from 2286 reflections
*a* = 11.785 (2) Å	θ = 3.1–27.5°
*b* = 12.716 (3) Å	µ = 0.27 mm^−^^1^
*c* = 13.288 (3) Å	*T* = 298 K
*V* = 1991.3 (7) Å^3^	Block, colourless
*Z* = 8	0.20 × 0.05 × 0.05 mm

### Data collection

**Table:**

Rigaku Mercury2 CCD diffractometer	2286 independent reflections
Radiation source: fine-focus sealed tube	1864 reflections with *I* > 2σ(*I*)
Graphite monochromator	*R*_int_ = 0.051
Detector resolution: 13.6612 pixels mm^-1^	θ_max_ = 27.5°, θ_min_ = 3.1°
CCD profile fitting scans	*h* = −15→15
Absorption correction: multi-scan (*CrystalClear*; Rigaku, 2005)	*k* = −16→16
*T*_min_ = 0.90, *T*_max_ = 1.00	*l* = −17→17
19260 measured reflections	

### Refinement

**Table:**

Refinement on *F*^2^	Secondary atom site location: difference Fourier map
Least-squares matrix: full	Hydrogen site location: inferred from neighbouring sites
*R*[*F*^2^ > 2σ(*F*^2^)] = 0.045	H-atom parameters constrained
*wR*(*F*^2^) = 0.107	*w* = 1/[σ^2^(*F*_o_^2^) + (0.0402*P*)^2^ + 0.4971*P*] where *P* = (*F*_o_^2^ + 2*F*_c_^2^)/3
*S* = 1.12	(Δ/σ)_max_ < 0.001
2286 reflections	Δρ_max_ = 0.18 e Å^−^^3^
96 parameters	Δρ_min_ = −0.15 e Å^−^^3^
0 restraints	Extinction correction: *SHELXL97* (Sheldrick, 2008), Fc^*^=kFc[1+0.001xFc^2^λ^3^/sin(2θ)]^-1/4^
Primary atom site location: structure-invariant direct methods	Extinction coefficient: 0.044 (3)

### Special details

**Table:**

Geometry. All esds (except the esd in the dihedral angle between two l.s. planes) are estimated using the full covariance matrix. The cell esds are taken into account individually in the estimation of esds in distances, angles and torsion angles; correlations between esds in cell parameters are only used when they are defined by crystal symmetry. An approximate (isotropic) treatment of cell esds is used for estimating esds involving l.s. planes.
Refinement. Refinement of F^2^ against ALL reflections. The weighted R-factor wR and goodness of fit S are based on F^2^, conventional R-factors R are based on F, with F set to zero for negative F^2^. The threshold expression of F^2^ > 2sigma(F^2^) is used only for calculating R-factors(gt) etc. and is not relevant to the choice of reflections for refinement. R-factors based on F^2^ are statistically about twice as large as those based on F, and R- factors based on ALL data will be even larger.

### Fractional atomic coordinates and isotropic or equivalent isotropic displacement parameters (Å^2^)

**Table:**

	*x*	*y*	*z*	*U*_iso_*/*U*_eq_	
N2	0.42899 (11)	0.17785 (9)	0.36893 (10)	0.0400 (3)	
H2D	0.3915	0.2112	0.3193	0.048*	
H2E	0.3769	0.1567	0.4141	0.048*	
C3	0.48334 (15)	0.08132 (13)	0.32473 (13)	0.0476 (4)	
H3A	0.5226	0.0434	0.3787	0.057*	
C5	0.57312 (16)	0.20499 (14)	0.50250 (15)	0.0551 (5)	
H5A	0.6217	0.1522	0.4741	0.083*	
H5B	0.5224	0.1729	0.5500	0.083*	
H5C	0.6184	0.2571	0.5361	0.083*	
C6	0.50507 (15)	0.25665 (13)	0.41958 (13)	0.0471 (4)	
H6A	0.5578	0.2850	0.3694	0.056*	
C7	0.4316 (2)	0.34550 (15)	0.45802 (16)	0.0666 (6)	
H7A	0.3883	0.3743	0.4033	0.100*	
H7B	0.4788	0.3994	0.4864	0.100*	
H7C	0.3809	0.3193	0.5087	0.100*	
C4	0.3906 (2)	0.01151 (17)	0.28384 (17)	0.0727 (6)	
H4A	0.4231	−0.0530	0.2598	0.109*	
H4B	0.3527	0.0467	0.2294	0.109*	
H4C	0.3369	−0.0036	0.3363	0.109*	
C2	0.5690 (2)	0.1114 (2)	0.24603 (18)	0.0774 (7)	
H2A	0.6292	0.1508	0.2768	0.116*	
H2B	0.5330	0.1536	0.1954	0.116*	
H2C	0.5996	0.0490	0.2157	0.116*	
S1	0.26970 (4)	0.06903 (3)	0.54981 (3)	0.04839 (18)	
N1	0.32617 (17)	0.20204 (15)	0.70857 (14)	0.0754 (6)	
C1	0.30276 (14)	0.14704 (14)	0.64316 (13)	0.0459 (4)	

### Atomic displacement parameters (Å^2^)

**Table:**

	*U*^11^	*U*^22^	*U*^33^	*U*^12^	*U*^13^	*U*^23^
N2	0.0425 (7)	0.0407 (7)	0.0369 (7)	0.0020 (6)	−0.0008 (6)	0.0052 (6)
C3	0.0555 (10)	0.0441 (9)	0.0432 (9)	0.0098 (8)	−0.0096 (8)	−0.0048 (7)
C5	0.0523 (10)	0.0544 (11)	0.0586 (11)	−0.0096 (8)	−0.0106 (9)	−0.0078 (9)
C6	0.0553 (10)	0.0370 (8)	0.0489 (9)	−0.0081 (7)	0.0062 (8)	0.0020 (7)
C7	0.0863 (15)	0.0420 (10)	0.0714 (14)	0.0048 (10)	0.0026 (11)	−0.0043 (9)
C4	0.0806 (15)	0.0675 (13)	0.0700 (14)	−0.0024 (11)	−0.0161 (11)	−0.0230 (11)
C2	0.0745 (15)	0.0887 (16)	0.0690 (14)	0.0145 (12)	0.0166 (11)	−0.0150 (13)
S1	0.0527 (3)	0.0463 (3)	0.0462 (3)	−0.00456 (19)	0.00129 (19)	−0.00236 (19)
N1	0.0879 (14)	0.0808 (12)	0.0576 (11)	−0.0223 (11)	−0.0029 (9)	−0.0157 (9)
C1	0.0422 (9)	0.0503 (10)	0.0451 (9)	−0.0037 (7)	0.0023 (7)	0.0037 (8)

### Geometric parameters (Å, º)

**Table:**

N2—C6	1.504 (2)	C6—H6A	0.9800
N2—C3	1.504 (2)	C7—H7A	0.9600
N2—H2D	0.9000	C7—H7B	0.9600
N2—H2E	0.9000	C7—H7C	0.9600
C3—C2	1.503 (3)	C4—H4A	0.9600
C3—C4	1.509 (3)	C4—H4B	0.9600
C3—H3A	0.9800	C4—H4C	0.9600
C5—C6	1.513 (2)	C2—H2A	0.9600
C5—H5A	0.9600	C2—H2B	0.9600
C5—H5B	0.9600	C2—H2C	0.9600
C5—H5C	0.9600	S1—C1	1.6354 (19)
C6—C7	1.512 (3)	N1—C1	1.149 (2)
			
C6—N2—C3	117.69 (13)	C7—C6—H6A	108.6
C6—N2—H2D	107.9	C5—C6—H6A	108.6
C3—N2—H2D	107.9	C6—C7—H7A	109.5
C6—N2—H2E	107.9	C6—C7—H7B	109.5
C3—N2—H2E	107.9	H7A—C7—H7B	109.5
H2D—N2—H2E	107.2	C6—C7—H7C	109.5
C2—C3—N2	110.49 (15)	H7A—C7—H7C	109.5
C2—C3—C4	112.70 (17)	H7B—C7—H7C	109.5
N2—C3—C4	108.19 (15)	C3—C4—H4A	109.5
C2—C3—H3A	108.5	C3—C4—H4B	109.5
N2—C3—H3A	108.5	H4A—C4—H4B	109.5
C4—C3—H3A	108.5	C3—C4—H4C	109.5
C6—C5—H5A	109.5	H4A—C4—H4C	109.5
C6—C5—H5B	109.5	H4B—C4—H4C	109.5
H5A—C5—H5B	109.5	C3—C2—H2A	109.5
C6—C5—H5C	109.5	C3—C2—H2B	109.5
H5A—C5—H5C	109.5	H2A—C2—H2B	109.5
H5B—C5—H5C	109.5	C3—C2—H2C	109.5
N2—C6—C7	107.91 (15)	H2A—C2—H2C	109.5
N2—C6—C5	110.65 (13)	H2B—C2—H2C	109.5
C7—C6—C5	112.46 (16)	N1—C1—S1	179.8 (2)
N2—C6—H6A	108.6		
			
C6—N2—C3—C2	−59.45 (19)	C3—N2—C6—C7	−179.73 (14)
C6—N2—C3—C4	176.74 (15)	C3—N2—C6—C5	−56.31 (19)

### Hydrogen-bond geometry (Å, º)

**Table:**

*D*—H···*A*	*D*—H	H···*A*	*D*···*A*	*D*—H···*A*
N2—H2*D*···N1^i^	0.90	1.99	2.888 (2)	172
N2—H2*E*···S1	0.90	2.47	3.3490 (14)	166

Symmetry code: (i) *x*, −*y*+1/2, *z*−1/2.
